# The emergence of synchrony in networks of mutually inferring neurons

**DOI:** 10.1038/s41598-019-42821-7

**Published:** 2019-04-30

**Authors:** Ensor Rafael Palacios, Takuya Isomura, Thomas Parr, Karl Friston

**Affiliations:** 1 The Wellcome Centre for Human Neuroimaging, University College London, Queen Square, London WC1N 3BG UK; 2 Laboratory for Neural Computation and Adaptation, RIKEN Center for Brain Science, Hirosawa, Wako, Saitama 351-0198 Japan

**Keywords:** Dynamical systems, Synaptic plasticity

## Abstract

This paper considers the emergence of a generalised synchrony in ensembles of coupled self-organising systems, such as neurons. We start from the premise that any self-organising system complies with the free energy principle, in virtue of placing an upper bound on its entropy. Crucially, the free energy principle allows one to interpret biological systems as inferring the state of their environment or external milieu. An emergent property of this inference is synchronisation among an ensemble of systems that infer each other. Here, we investigate the implications of neuronal dynamics by simulating neuronal networks, where each neuron minimises its free energy. We cast the ensuing ensemble dynamics in terms of inference and show that cardinal behaviours of neuronal networks – both *in vivo* and *in vitro* – can be explained by this framework. In particular, we test the hypotheses that (i) generalised synchrony is an emergent property of free energy minimisation; thereby explaining synchronisation in the resting brain: (ii) desynchronisation is induced by exogenous input; thereby explaining event-related desynchronisation and (iii) structure learning emerges in response to causal structure in exogenous input; thereby explaining functional segregation in real neuronal systems.

## Introduction

Any biological or self-organising system is characterised by the ability to maintain itself in a changing environment. The separation of a system from its environment – and the implicit delineation of its boundaries – mandates autopoietic, autonomous behaviour^1^. This rests on the capacity to resist the tendency of increasing disorder or entropy entailed by the second law of thermodynamics, or more exactly the fluctuation theorems for non-equilibrium systems^2^. Given the phase space of all possible states a system can occupy, a subspace can be identified that comprises the most likely states in which a self-organising system is found. In other words, the system’s (random) dynamics can be described as an itinerant orbit in phase space that keeps revisiting the same states (c.f., homeostasis). These states are called a pullback or random global attractor. This attracting set has an associated probability distribution, which is the probability of the system being found in any state; namely, the ergodic density^3,4^. Generally, the ergodic density of biological systems will have a low entropy, as the probability over states is concentrated within a small volume, compared to the volume of the entire state space (i.e., its characteristic states that are within some physiological bounds).

The formulation of biological self-organisation in terms of density dynamics allows one to describe self-organisation in terms of information theory or systems that minimise variational free energy^1^. In brief, the free energy principle treats a system as a probabilistic model of its environment that minimises an upper bound on the self-information or surprise (i.e., negative log marginal likelihood) of sensations; referred to as variational free energy. From a statistical (information theoretic) perspective, this is equivalent to saying that the system maximises model evidence, which can be interpreted in terms of making inferences. Because Shannon entropy is the long-term average of surprise, under ergodic assumptions, bounding surprise is the same of limiting the dispersion or entropy of sensory states^5^. Therefore, a system that minimises variational free energy must be a self-organising, ergodic system endowed with a random global attractor. Our focus here is upon self-organization in neuronal systems, i.e., a neuronal network.

Neuronal dynamics can be governed by different types of attractors, including point attractors, periodic orbits, quasi-periodic orbits, and chaotic dynamics (heteroclinic channels)^6,7^. These attractors exhibit qualitatively distinct characteristics; nonetheless, any neuronal system that has an attractor, and thereby minimises free energy, must maintain some sort of synchrony with its environment^8^. This is a property of any open self-organising system, which can be understood in terms of the exchange between the internal and external states of a system^9^. This exchange rests on the existence of a set of states that are internal to the system (e.g. cytoplasm within a cell, neurons within the brain), a set of external states (e.g. the extracellular environment or the world), and a third set of boundary states that separates them called a Markov blanket^1,10^. Crucially, the free energy formulation licenses an interpretation of internal states as instantiating probabilistic beliefs about external states. In other words, internal states parameterise a probability density over external states and can therefore be interpreted in terms of an elemental form of inference. This follows because internal dynamics can be expressed as a gradient flow on variational free energy. Minimising variational free energy implies that posterior or Bayesian beliefs encoded by internal states become a Bayes optimal representation of external states^5^. In what follows, we will use this interpretation of dynamics to talk about the beliefs (used in the sense of belief propagation and Bayesian belief updating – as opposed to propositional or subjective beliefs) of a neuron about its external states, which are the Markov blanket states of other neurons. Heuristically, this means that a network of coupled neurons will try to infer the states of every other neuron in the network.

In summary, the internal and environmental dynamics of any self-organising system become coupled as free energy is minimised, which enforces a (generalised) synchrony between internal dynamics and external states^8^. When extending this picture to ensembles of coupled self-organising systems, such as neurons, the internal states of one system become the external states of another: this means the entire state space of the ensemble can be partitioned into an ensemble of Markov blankets and the internal states each blanket surrounds^11^. Consequently, the synchronisation implied by free energy minimisation emerges among the internal states that are coupled via their Markov blankets^12^.

Here, we leverage the deep connection between free energy principle, self-organisation and synchrony to investigate synchronisation in neuronal networks comprising free energy minimising neurons. In particular, we ask whether the single principle of minimising variational free energy can account for some ubiquitous characteristics of real neuronal networks; both *in vivo* and *in vitro*. These characteristics include the emergence of (identical) generalised synchronisation at particular frequencies; for example, the ubiquitous alpha frequency found in the resting brain^13–15^. When stimulated or activated by sensory input, real neuronal ensembles typically desynchronise, showing faster high-frequency dynamics^16–18^. Finally, neuronal dynamics are characteristically itinerant and structured^19–22^ showing a segregation of synchronisation during neurodevelopment and sensory learning^23,24^. This segregation is often associated with functional specialisation; for example, the celebrated what and where pathways in the ventral and dorsal streams of the visual hierarchy^25^.

Among the variety of attractors, oscillatory fluctuations are observed at all levels of the nervous system, from the intracellular level to the global network. For example, at the single neuronal level, the membrane potential can change with relatively constant period^26^, while oscillations are a hallmark of mesoscopic (e.g. local-field potentials) and macroscopic (e.g. electroencephalography and magnetoencephalography) fluctuations^27^. Intrinsic oscillators play a crucial role in the brain as central pattern generators in the motor system^28^ and cortical dynamics^29,30^. Under the free energy principle, intrinsic oscillations become the sort of dynamics a neuron expects to encounter: neurons have (genetically encoded) beliefs that the cause of excitatory post-synaptic potentials (EPSPs) follow a certain pattern^31^. In what follows, we will use simulations of neural networks to illustrate that (e.g. alpha) synchronisation; event related desynchronisation and functional specialisation are all emergent properties of coupled neurons under the following two assumptions:

- Neuronal dynamics and plasticity minimise variational free energy, under a simple generative model.
- This generative model entails the prior belief that a neuron’s presynaptic inputs are generated by an external state with a quasi-periodic orbit.

Put more simply, all that we will assume is that neurons are equipped with the prior belief that to be a neuron means to participate in a network that has an attracting set (i.e., a quasi-periodic orbit).

The simulations used to illustrate the emergent properties use a biologically plausible integration scheme that can be described in terms of synaptic activity, plasticity and homoeostasis. This scheme has been applied in several domains; including, the simulation of *in vivo*^32^ and *in vitro* neuronal networks^33^. We will consider all three timescales of free energy minimisation, where synaptic activity corresponds to a gradient flow on variational free energy^32,34^, while slower changes in synaptic efficacy (i.e., strength or precision) perform a gradient descent on variational free energy^35^. Finally, synaptic homoeostasis is implemented using Bayesian model selection, eliminating (redundant) synaptic connections to minimise the variational free energy associated with neuronal connections^36,37^. Note that these three processes optimise exactly the same quantity – namely, variational free energy over different timescales – in a way that is very similar to real neuronal dynamics, plasticity and synaptic elimination.

In summary, this paper offers *in silico* demonstrations of self-organisation in neuronal networks of coupled intrinsic oscillators. First, we consider how neuronal dynamics and plasticity can be formulated as variational free minimisation – and explain the implicit (Bayesian) inference process or model inversion. Next, we perform numerical simulations of coupled neurons, in which each neuron is, effectively, inferring states of its colleagues, while undergoing short-term synaptic plasticity and pruning to adapt to their presynaptic inputs. These simulations illustrate the emergence of synchronisation, desynchronisation and functional specialisation. Finally, we conclude with a discussion of biological interpretations of the simulation results.

## Free Energy Minimisation and Variational Message Passing In Neurons

A tenet of the free energy principle is that any biological system has to minimise the surprise associated with sensory input or, equivalently, to minimise variational free energy^5^. Minimising free energy maximises the evidence for a probabilistic or generative model of how observations $\tilde{o}=({o}_{1},{o}_{2},\ldots ,{o}_{t})$ are caused by external states $\tilde{s}=({s}_{1},{s}_{2},\ldots ,{s}_{t})$ and parameters *θ* of the external world. Here *o*_*τ*_ and *s*_*τ*_ indicate the observation and external state at time *τ*, respectively. The variational free energy depends on generative $P(\tilde{o},\tilde{s},\theta |m)$ and recognition densities $Q(\tilde{s},\theta )=\prod _{\tau =1}^{t}Q({s}_{\tau })Q(\theta ),$ expressing the prior and posterior beliefs of the system about external states and parameters of the world.

In this construction, the posterior beliefs are parameterised by the internal states of the system and the variational free energy is a functional of these beliefs and observations (i.e., presynaptic inputs generated by other neurons that constitute a single neuron’s external states):

1 $$\begin{array}{c}F\equiv {\sum }_{\tau =1}^{t}{{\rm{E}}}_{Q({s}_{\tau })Q({s}_{\tau -1})Q(\theta )}[-\mathrm{ln}\,P({o}_{\tau }|{s}_{\tau },\theta ,m)-\,\mathrm{ln}\,P({s}_{\tau }|{s}_{\tau -1},\theta ,m)+\,\mathrm{ln}\,Q({s}_{\tau })]\\ \,\,\,\,\,\,\,\,\,\,+\,{{\rm{E}}}_{Q(\theta )}[-\,\mathrm{ln}\,P(\theta |m)+\,\mathrm{ln}\,Q(\theta )]\end{array}$$

Neuronal dynamics and synaptic plasticity can be cast in terms of an inference or evidence accumulation by minimising this functional with respect to internal states and parameters (encoding the posterior beliefs). This means the resulting dynamics and plasticity depend only upon the generative model^34^. Although neuronal dynamics are usually described in terms of differential equations^32,38^, the generative model employed here is discrete. The justification for this is that we model spiking neurons, whose inference process predicts the generation of discrete action potentials.

**Figure 1:**
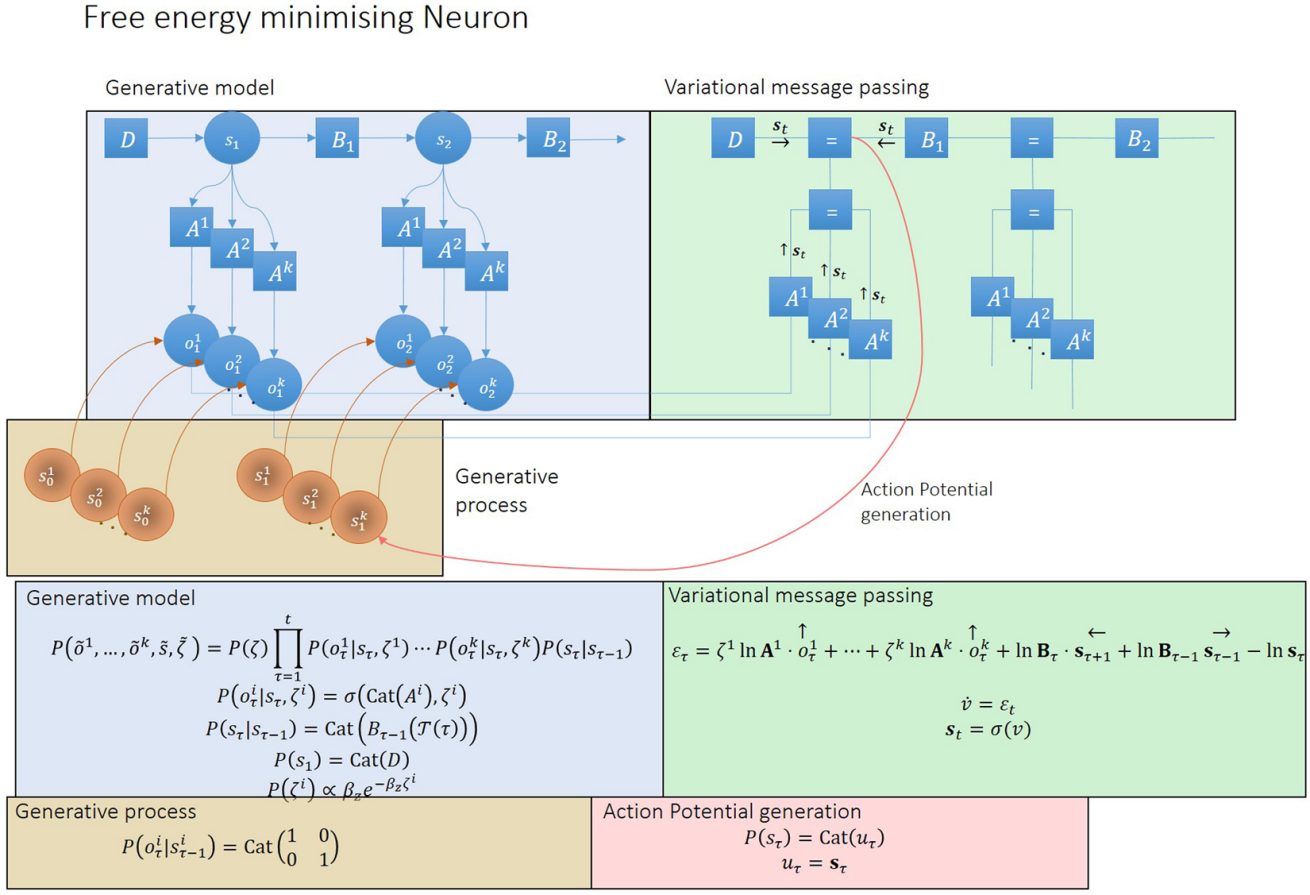
Schematics that illustrate the generative process and model. Blue upper panel: This panel illustrates a probabilistic graphical model from which observations are generated. The circles indicate hidden or sensory states (random variables); squares, the probability distribution functions generating these states; and arrows, the causal relationships linking functions to states. Blue bottom left panel: mathematical description of the generative model. The first equality specifies the form of the generative model (neuronal beliefs about the causal relationship between hidden causes and observations). This can be expressed in terms of *A*, *D* and *B* matrices, defining the probability of observing EPSPs given the network state, the probability of the initial network state and the probabilistic state transitions respectively. Each *A* matrix or connection between network and neuron has an associated precision, representing its synaptic strength (that the neuron has gamma prior beliefs about). Brown upper panel: the generative model is different from the real process generating EPSPs; as observations are caused by multiple neurons, instead of by one neuronal network. Brown lower panel: each pre-synaptic neuron is a hidden state ${s}_{t-1}^{i}$ – representing its state of firing – causing one post-synaptic observation ${o}_{t}^{i}$. Green upper panel: In this Forney factor graph, squares represent factors or conditional probabilities over random variables (hidden states); edges correspond to the (marginal probability densities over) random variables that are passed between nodes; and equal signs link edges instantiating the same random variable. This schematic nicely summarises the process of model inversion; as it shows the convergence (equal signs) of messages (edges) coming from all factors (squares) contributing to the inference of the same random variable. Green lower panel: the messages are compared to the actual belief about the network hidden state. The resulting prediction error is then used to update this belief. Red lower panel: once prediction error has been minimised, the neuron decides whether to generate or not an action potential *s*_*t*_ based on the updated belief *s*_*t*_, which then feeds back to the network (red arrow), generating a new observation at *t* + 1.

In our setup, each neuron acts as both the process generating observations – for other neurons – and the generative model that infers the cause of its own observations. Suppose a network comprises *n* neurons. As a generative process, neuron *i* becomes a discrete, one-dimensional hidden cause ${s}_{\tau -1}^{i}\in \{0,1\}$ that can be either in a silent (0) or firing (1) state. If it is firing, the pre-synaptic neuron *i* causes a single EPSP at the subsequent time *τ* in a post-synaptic neuron $i^{\prime} =1,2,\ldots ,n$ (upper brown panel in Fig. 1). The EPSP becomes the sensory input or observation $({o}_{\tau }^{i})$ for the post-synaptic neuron. In terms of inference, each neuron processes a continuous, one-dimensional internal state ${{\bf{s}}}_{\tau }\in [0,1]$ that encodes its belief (i.e. expectation) about the hidden state of the external world. Here, this hidden state represents the expected state of the network; i.e., the average propensity of a neuron to fire. The *i*-th neuron then generates action in a form of discrete outcome ${o}_{\tau +1}^{i}\in \{0,1\}$ at time *τ* + 1, with $i=1,2,\ldots ,n$ synapses. Note that the action of one neuron constitutes the observed outcomes for another (at the subsequent time step). A neuron therefore fires with a probability that corresponds to its beliefs about the state of the network in which it participates. Synaptic connections are represented internally by neuronal parameters in terms of a likelihood or *A* matrix, which encodes the probability of observing a particular ${o}_{\tau }^{i}$ given *s*_*τ*_. The ensuing generative model is shown in Fig. 1 (upper light blue panel) as a probabilistic graphical model, which takes the form of a hidden Markov model^34^. In this generative model, the network state depends only on the previous state *s*_*τ* − 1_ (actually use a semi-Markovian process as described below) and the current observations. The conditional dependence of *s*_*τ*_ on *s*_*τ* − 1_ is then parameterised by a transition or *B* matrix.

The generative process for neuronal states differs from the neuronal beliefs about this process: neurons encode the network as a single hidden state, generating high dimensional observations, via different connections. In reality, the network comprises many neurons, each generating its own outputs, conveyed through distinct synapses (upper brown panel in Fig. 1). Neurons are therefore encoding the expected state of the network by averaging over the EPSPs impinging on them, through spatial summation. Formally speaking, this is a mean field approximation, as the interactions among pre-synaptic neurons are treated as negligible fluctuations around a mean interaction between the network and the post-synaptic neuron. We now look more closely at the neuronal dynamics and plasticity implied by the minimisation of variational free energy under this minimal setup.

### Variational message passing

Interpreting neuronal dynamics in terms of model inversion enables us to associate neuronal processes with inference about external states and learning of model parameters. In the following, we first focus on neuronal dynamics and inference. The requisite inversion scheme – for discrete models – rests upon variational message passing of the sort used in approximate Bayesian inference. This message passing is illustrated in the Forney factor graph^39^ in Fig. 1 (upper right green panel).

The associated belief update equations are shown in the lower green panel of Fig. 1 (the derivations of these equations are described in Methods). Inference about the external state at each moment in time depends on messages derived from present observations, a forward message or prediction and a backward message or postdiction. Observations consist of EPSPs that sum at the level of the soma, whereas predictions impose an activity-dependent modulation; e.g. frequency adaptation driven by the recent neuronal activity history^40^ on the biophysical machinery (e.g. voltage-gated sodium channels) controlling the voltage at the soma (in particular, near the axon hillock). We can ignore the postdiction in our modelling of neuronal dynamics, as it plays a negligible role in the inference process (postdiction is informed by expectations about hidden states in the future, which in turn rely on beliefs about the policy or course of action a system entertains, which are not part of the neuronal generative model).

At each time point, these messages converge, driving a change in the log expectation of the external state via an error term. This log expectation can be thought of as the voltage at the neuron’s soma, and its change as depolarisation. This renders the softmax operator equivalent to a (sigmoid) firing rate-depolarisation activation function, and the associated expectation the instantaneous firing rate^34^. In other words, a neuron’s belief about the state of the network is encoded in the probability of firing an action potential. Unbeknown to itself, it broadcasts this belief – by emitting an action potential that serves as a presynaptic input to other neurons. In the formalism of Fig. 1, each neuron’s action (i.e., firing or not firing) *μ*_*t*_ is drawn from a Bernoulli distribution that reflects its expectation *u*_*τ*_ = **s**_*t*_ that the network – in which it is participating – is currently firing (red panel in Fig. 1). Usually, in simulations of neural networks that perform variational message passing, one would model a neuronal population; such that the population firing rate was a continuous variable. However, here, we are considering each neuron as a free energy minimising entity – and neuronal firing becomes a discrete action on external states (i.e., the states of other neurons).

As noted in the introduction, the reciprocal coupling between a system and its environment underwrites the emergence of synchronisation in ensembles of self-organising systems^19,41–46^. This generalised synchrony becomes especially transparent under free energy minimisation: this is because the best way to predict the behaviour of a system’like me’ is to behave exactly ‘like you’ so that we can both predict ‘each other’. See^8^ for an illustration of this in the context of communication and continuous time generative models. In other words, a system tries to explain the world it inhabits, while acting to make the world consistent with its explanations (i.e. active inference).

Notice that, in the current setting, action potential firing simply reports the ‘beliefs’ of a neuron about external state of affairs (i.e., whether it is part of an ensemble that is firing or not). This differs from the usual treatment of action in active inference, where an action is selected – based on a generative model – to realise some predicted consequences of action. However, in the current setup, things are much simpler, because the external milieu comprises other neurons that share a common generative model. This means the action of neurons (i.e., whether they are firing or not) is a consequence of their inference about whether neurons are firing (or not). This means that it is sufficient to broadcast ‘beliefs’ to realise each neuron’s predictions. Heuristically, the free energy minimising solution obtains when all neurons believe they are firing (or not) and report their ‘beliefs’ by firing (or not); thereby providing evidence for other neurons that they are firing (or not). This solution underwrites the generalised synchronisation and distributed activity illustrated above. When the neurons are sufficiently similar, their synchronisation manifold (i.e., the random dynamical attractor for the ensemble) becomes an identical synchronisation manifold. In this setting, all coupled neurons come to oscillate in synchrony. This is remarkably similar to experimental observations that a network of neurons exhibits a synchronous oscillation, even when they oscillate with different intrinsic frequencies in isolation^47^. From the viewpoint of Bayesian inference, this identical synchronisation is a natural consequence of mutual inference – that minimises the variational free energy of each constituent neuron and, implicitly, the variational free energy of the ensemble.

## Simulation and Results

In this section, we provide a numerical analysis to illustrate the emergence of synchronous oscillatory activity in neuronal networks as a consequence of mutual inference according to free energy minimisation. This illustration requires us to specify the generative model for each neuron, which, as noted in the introduction, reflects each neuron’s belief that it is embedded in an oscillatory network.

### Intrinsic oscillatory neurons

We first characterised self-organisation in the context of oscillatory neurons. One can simulate oscillatory behaviour via a generative model that instantiates the belief that network dynamics possess an orbit. This model determines subsequent belief updating and neuronal dynamics (i.e., postsynaptic responses and ensuing action or neuronal firing). As noted above, we used a simple generative model that does not entertain beliefs about sequences of states. In other words, only probability transitions over one time step are considered. Therefore, to generate nontrivial orbits, we used a semi-Markovian model of state transitions, whereby inference about the present depends on the history of previous states: ${\mathscr{T}}(\tau )$. Here, each element of *B*_*τ*_ denotes a transition probability from the state at *τ* − 1 to the state at *τ*, ${({B}_{\tau })}_{ij}\equiv P({s}_{\tau }=i|{s}_{\tau -1}=j,{B}_{\tau -1}({\mathscr{T}}(\tau )))$. In short, we define probabilistic state transitions in a manner that depends on the recent history of observations:

2 $${B}_{\tau }({\mathscr{T}}(\tau ))\equiv P({s}_{\tau }|{s}_{\tau -1},{B}_{\tau -1}({\mathscr{T}}(\tau )))=\{\begin{array}{l}(\begin{array}{cc}0 & 0\\ 1 & 1\end{array})\,{\rm{If}}\,{\sum }_{\tau ^{\prime} \in {\mathscr{T}}(\tau )}{s}_{\tau ^{\prime} }=0\\ (\begin{array}{cc}1 & 1\\ 0 & 0\end{array})\,{\rm{Otherwise}}\,\end{array}$$

$${\mathscr{T}}(\tau )\equiv \{\tau -{t}_{{\rm{\max }}},\tau -{t}_{{\rm{\max }}}+1,\ldots ,\tau -{t}_{{\rm{\min }}}\}\,{\rm{where}}\,{t}_{{\rm{\max }}} > {t}_{{\rm{\min }}}$$

Equation 2 expresses the prior expectation that the network is firing (i.e. *s*_*τ*_ = 1) if it has been silent during the interval ${\mathscr{T}}(\tau )$, and that it is silent (i.e. *s*_*τ*_ = 0) otherwise. The history for a neuron depends on the sequence of internal states over the period ${\mathscr{T}}$; because the instantaneous firing rate is a continuous variable, a neuron is considered to have fired if its instantaneous firing rate at that moment was above chance and silent otherwise. The present moment is indicated by *τ*, whereas *t*_max_ and *t*_min_ delimit the temporal window of past expectations. Effectively, the interval ${\mathscr{T}}(\tau )$ corresponds to an inter-spike interval. The interval from *t*_min_ to *τ* does not enter the transition probabilities and, effectively, allows the network to fire consecutively (i.e., burst)^21^.

This formulation of the transition matrix is unusual and serves the purpose of accommodating conditional dependencies of the present state on an extended period or history. Technically, the state-dependent *B* matrix is an approximation of an extended, high dimensional transition matrix $P({s}_{\tau }|{s}_{\tau -1},\ldots ,{s}_{1},{B}_{\tau -1})\approx P({s}_{\tau }|{s}_{\tau -1},{B}_{\tau -1}({\mathscr{T}}(\tau )))$, which allows us to express a deep temporal generative model while retaining the (semi) Markovian property.

### Simulated synaptic plasticity

**Figure 2:**
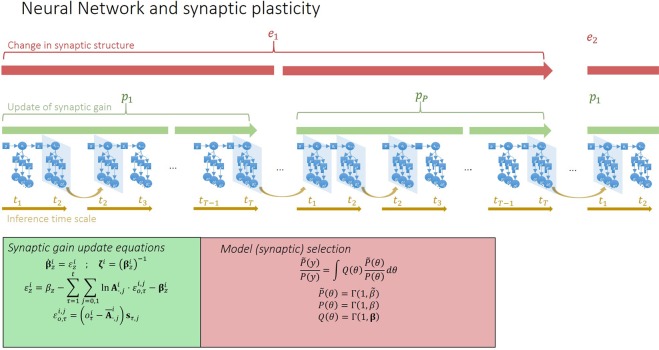
In the upper row, the time scales of neuronal dynamics are depicted by straight lines. These represent different aspects of neuronal processing. Specifically, brown arrows refer to inference about hidden states, which is associated with depolarisation in the soma and subsequent neuronal firing; green arrows represent the temporal scales of updating synaptic efficacy or short-term plasticity; and red arrows indicate time considered for *post hoc* structure learning or Bayesian model reduction. Brown curved arrows indicate the procedure used to simulate – as a continuous process – multiple neurons or inference machines comprising the network. The green and red panels at the bottom provide the equations that describe synaptic efficacy updates and Bayesian model reduction, respectively. Over a period *p*, an error term is accumulated at each synapse, which is then used to recalibrate synaptic strength, by changing the precision of the ***A*** matrix. This occurs multiple times over an epoch *e*, at the end of which the expected value *ζ* of the gain parameter for each synapse is used to calculate the Bayes factor $\,\frac{\tilde{P}(y)}{P(y)}$ for the full *P*(*y*) and reduced $\,\tilde{P}(y)$ model, with and without a particular synapse respectively. Here *y* indicates a set of the external states. The reduced model is accepted if the log Bayes factor is greater than 2.5. This is equivalent to saying that a neuron prunes a synapse if free energy would have been minimised to a greater extent had the neuron used a model without that particular parameter, with a margin of error less than 5% (i.e. if sensory data had afforded the reduced model roughly 20 times the evidence of the full model).

Neuronal dynamics unfold over many time scales, from fast voltage fluctuations in dendrites, to the ontological evolution of network structure. Here, we consider two types of dynamics in addition to somatic depolarisation, short-term plasticity and synaptic pruning:

Following previous work using continuous^48^ and discrete^32^ models, we consider fast changes in synaptic efficacy in terms of the deployment of attentional resources, which is usually associated with short-term synaptic plasticity. In the setting of neuronal inference, this is implemented as a synaptic-specific tuning of precision or gain of prediction errors. Hence, short-term plasticity can be associated with a (time-independent) precision parameter *ζ* that scales the likelihood *A* matrix, which plays the role of a connectivity or directed adjacency matrix. Heuristically, each neuron learns which synapses convey more precise observations about the state of the network, which in turn lead to more precise predictions. This process has a temporal scale, unfolding over *p* time steps (straight green arrow in Fig. 2). The requisite update equations are shown in the bottom left green panel in Fig. 2. In this formulation, each synapse (i.e., element of *A*) has an associated error term, which is accumulated over *p* time steps. This prediction error is then used to update the synaptic efficacies (see Methods for derivations).

**Figure 3:**
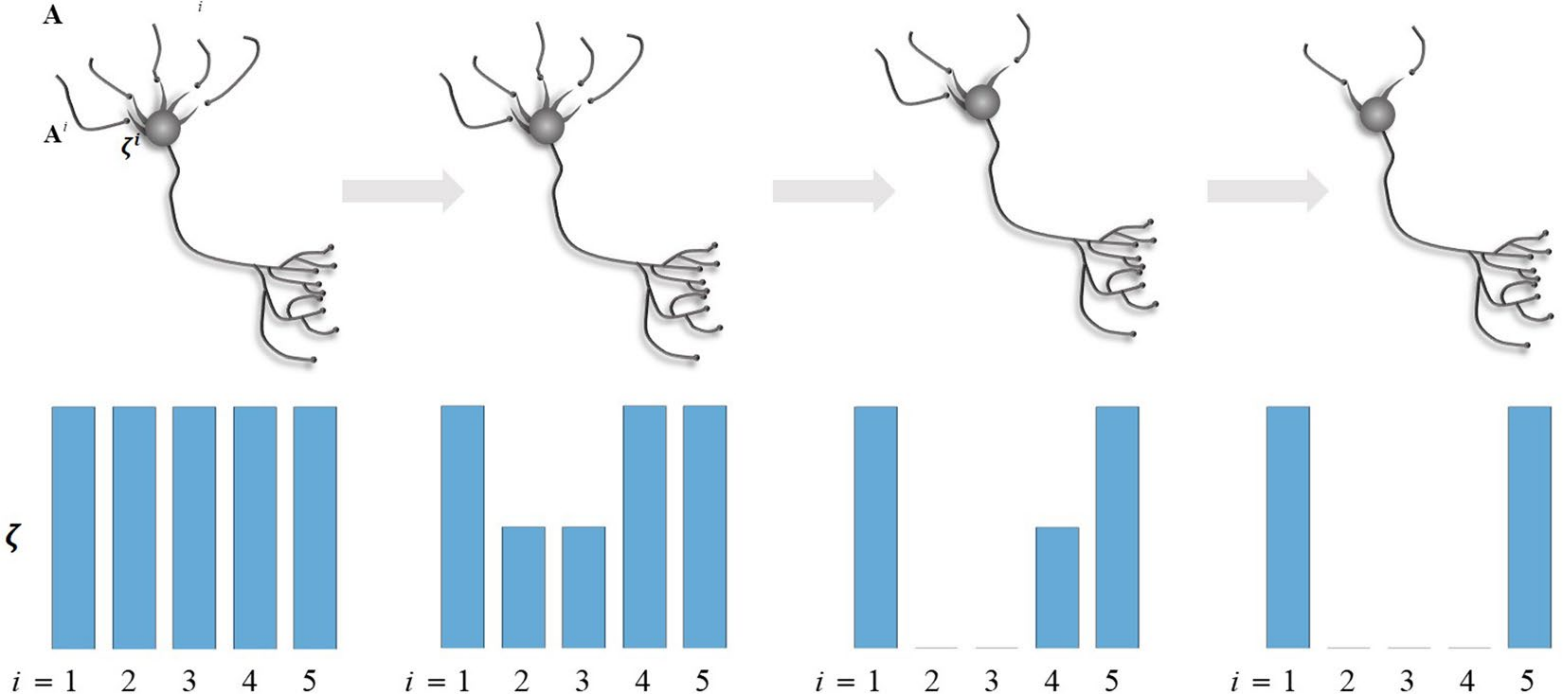
Schematic of a neuron connected to 5 presynaptic neurons. Each connection corresponds to an *A* matrix along with a precision term *ζ*. Over Epochs (grey arrows), neuronal connectivity structure changes based on the Bayes factor evaluated for each synapse: if the value of *ζ* drops to a value where the accuracy afforded by retaining a synapse does not compensate for its complexity cost, then the synapse is pruned.

At an even slower timescale, structure learning^49^, via synaptic pruning, constitutes another form of learning. At this timescale, variational free energy is not minimised with respect to synaptic efficacy but the structure of connectivity, as parameterised in terms of allowable synaptic connections. Each synapse is represented by a parameter of the generative model. Reducing the complexity of a model, while preserving accurate predictions, is an important part of free energy minimisation. In brief, this ensures the removal of redundant parameterisations and underwrites the capacity of a generative model to generalise to new data. In statistical terms, structure learning via Bayesian model selection precludes overfitting^5^. Synaptic pruning can therefore be cast in terms of refining model structure^50^. In the present setting, this entails a modification of beliefs about the relationship between the state *s*_*τ*_ of the network and the *i*^*th*^ observations $\,{o}_{\tau }^{i}$, parametrised by *ζ* ^*i*^ of the *i*^*th*^ synapse. Structure learning occurs over epochs *e* (red arrow in Fig. 2), and is implemented as *post hoc* Bayesian model selection^50^. This model is synaptic regression; whereby a reduced model (without a particular parameter or synapse) is compared with the full model (with that parameter), using the difference in model evidence or log Bayes factor (red panel in Fig. 2). A synapse is pruned if the accuracy – afforded by the parameter it encodes – does not sufficiently compensate for its complexity cost. A schematic of this process is illustrated in Fig. 3.

### Demonstration of emergent synchrony

Under the above setup, our simulations begin with a fully connected network of 16 neurons (excluding self-connections). Despite this small number of neurons, the ensuing dynamics bear a remarkable resemblance to experimental observations^51^ and theoretical treatments of intrinsic oscillatory neurons^52^. As noted above, neuronal dynamics are discrete, where we consider a time bin to represent an absolute refractory period of about 2 milliseconds. The parameters used in these simulations were chosen somewhat arbitrarily – our focus was on the interplay between key parameters that underwrite the attractor manifold that shapes network dynamics.

**Figure 4:**
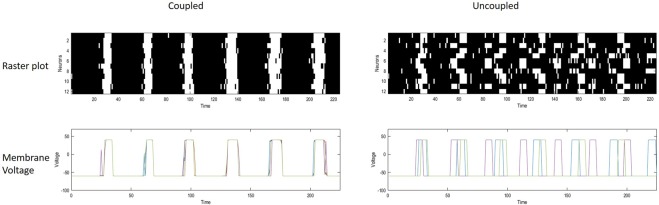
Networks of coupled and uncoupled neurons are compared. Upper panels: raster plots, with time and neuron on the x and y axis respectively. These panels show the firing of action potentials (in white) in the two conditions. Neurons have different intrinsic oscillatory periods; nonetheless, when they are coupled (left raster plot) they maintain a synchronised rhythmic activity throughout the simulation. On the contrary, when connections are disrupted, say by lowering [Ca^2+^], neurons rapidly desynchronise, and activity is noisier. The stochastic nature of neuronal dynamics comes from the absence of reliable observations, which makes beliefs about hidden states imprecise, and consequently the probability of firing (instantaneous firing rate) closer to chance levels. At the bottom, panels on both sides show variations in transmembrane potential at the soma over time. Each neuron is plotted with a line of different colour. In the coupled condition, lines are superimposed – because oscillations are synchronised over neurons, whereas on the right voltage fluctuations are clearly distinguishable.

We first compared networks of coupled and uncoupled neurons; namely, networks in which the coupling between neurons is switched on or off. To simulate a network of uncoupled neurons we set the precision of the *A* matrix to zero (Fig. 4). This could correspond to an attenuation of neurotransmitter release by manipulations of intracellular [Ca^2+^]. Neurons can express different intrinsic oscillatory periods, depending on model parameters; particularly synaptic precision. Here, we modelled inter-burst intervals ranging from 40 to 60 ms, and a bursting period extending up to 10 ms. The value of synaptic precision *ζ*, here set to 0.5, is particularly relevant in relation to the size of the network. This is because synaptic precision determines the influence of external inputs on neuronal belief updating, and consequently the relative sensitivity to intrinsic dynamics. These choices lend ensemble dynamics a characteristic timescale that reproduced the sort of fluctuations seen empirically: when uncoupled, the network rapidly desynchronises, whereas coupling among neurons restores a state of synchrony (Fig. 4). Moreover, an increase or decrease in the variability of autonomous firing rates can produce greater synchronisation or desynchronisation, respectively – provided synaptic weights are scaled appropriately.

**Figure 5:**
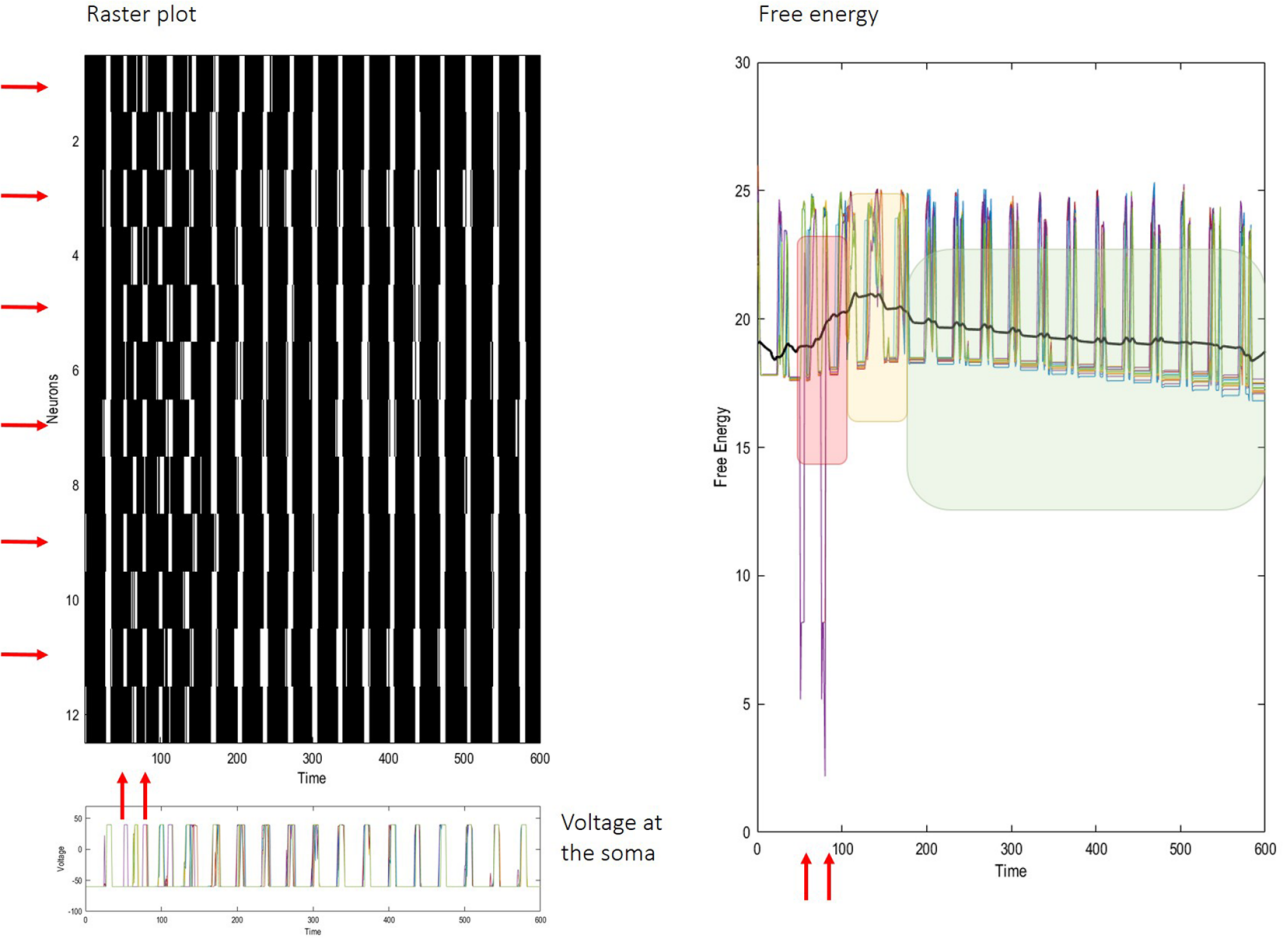
The raster plot on the top left shows network’s dynamics before, during, and after electrical stimulation of half of the neurons (indicated by the horizontal red arrows) at about time steps 50 and 75 of the simulation (vertical red arrows). External inputs cause part of the network to fire out of phase, but neurons quickly resynchronise within 150 ms. The membrane voltage at the soma is shown beneath the rasters. The right panel shows free energy for each neuron (coloured lines) and the average free energy (bold black line) over a sliding window of about 30 time steps. The red shade highlights increase in free energy due to the stimulation (vertical red arrows). The yellow shade focus on the rapid return to pre-stimulation levels, due to fast network resynchronisation. The green shade underscores the relatively slower temporal scale over which free energy is minimised due to short-term plasticity, which reflects the strengthening of connections between synchronised neurons. Notice that the free energy never approaches zero. This is because connections are never too precise; only the activation in concert of many synapses can have a noticeable effect on the post-synaptic neuron. This always entails a certain amount of uncertainty about the (hidden) network state.

We subsequently investigated how these neuronal networks respond to an external electrical stimulation (Fig. 5). Neurons were again equipped with varying intrinsic frequencies. We then administered two out-of-phase pulses to one half of the ensemble. Practically speaking, this was implemented by manipulating the ‘observations’ of a neuron, to mimic stimulation of the neuronal network at the appropriate time, and thereby induce neuronal responses. This sort of stimulation desynchronises network activity; however, neurons promptly return to a pre-stimulation state, as their activity converges to the synchronisation manifold (left panel). Relaxation to this attracting set is reflected in the changes in free energy – where the oscillatory dynamics correspond to a free energy minimum. The accompanying minimisation of variational free energy can be expressed over different time scales; based on the dynamic or plastic processes in play (see Fig. 5: right panel). Specifically, the synaptic weight was set to 0.5 at the beginning of each simulation. As the network recovers from a perturbation – and neurons come to predict each other – reciprocal connections steadily increase, up to 0.7 (at the end of simulation). This plasticity speaks to the potential importance of synaptic homoeostasis (not implemented here) to preclude runaway excitation. Here, the precision *ζ* afforded each synapse is in the order of thousandth, which ensures a smooth calibration of synaptic strength. In summary, this *in-silico* illustration shows that the dynamics of our neuronal network, characterised by an emergence of synchronisation, is resilient and capable of responding sensitively – and recovering from – following external perturbations. This is reminiscent of the characteristic desynchronisation seen in response to stimuli (a.k.a. electrophysiological activation)^17,18^. Furthermore, it is consistent with reappearance of alpha waves (i.e., slow synchronisation) upon eye closure observed in EEG studies^53^. In terms of free energy minimisation, one would interpret these instances of chaotic itinerancy as a relaxation to an attracting set, after external perturbations (e.g., thalamic input) are removed^22,54–56^.

So far, we have used a fully connected network that might be appropriate for small areas of the cortical sheet with dense intrinsic connections. However, macroscopic neuronal networks are characterised by a sparse connectivity in terms of the hierarchy and functional segregation^23,57–59^. To illustrate the emergence of sparse connectivity, via synaptic regression, we now call upon the free energy minimising processes associated with structure learning to simulate the emergence of functional specialisation.

**Figure 6:**
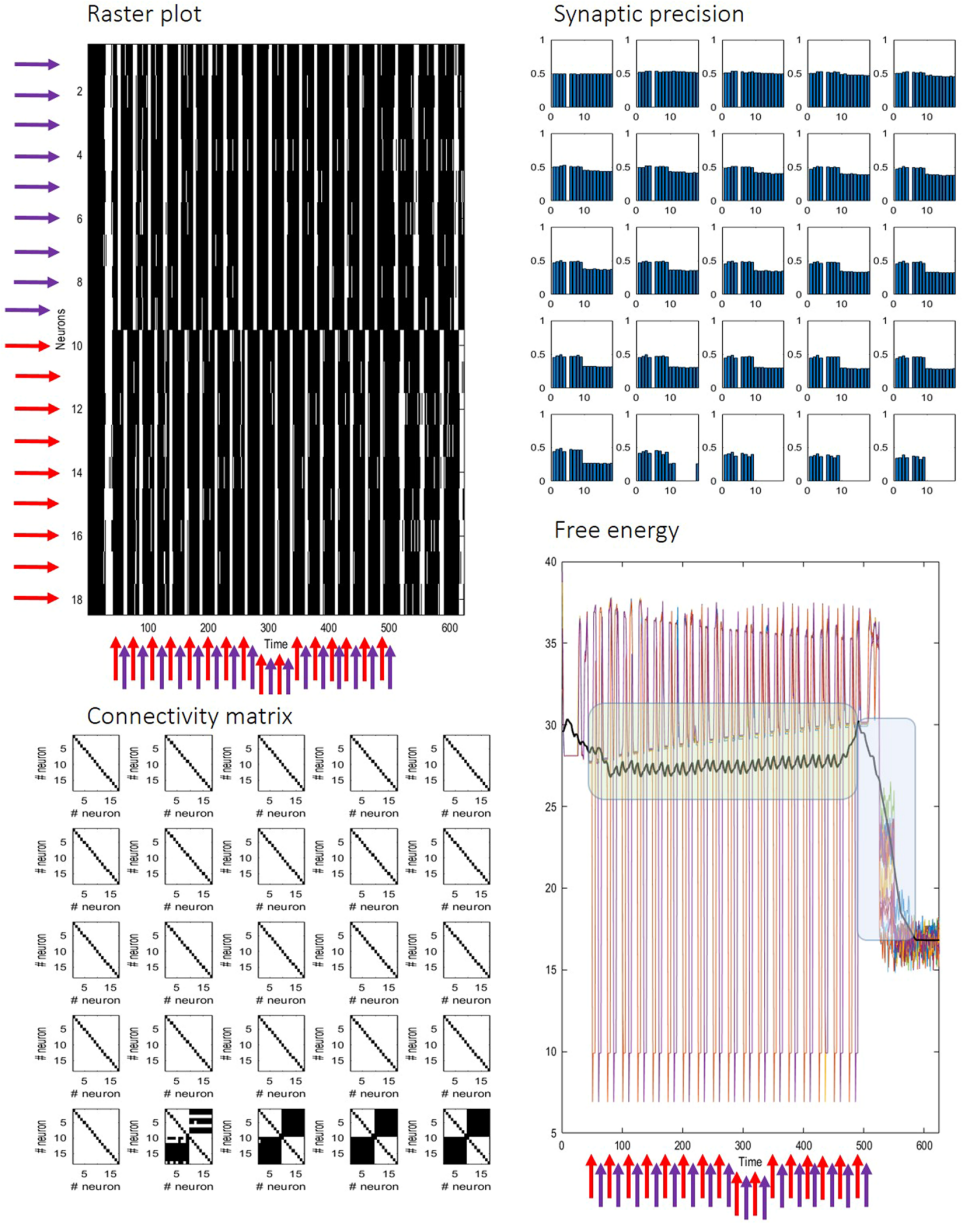
On the top left, the raster plot highlights the segregation of network dynamics caused by the external excitatory inputs (vertical arrows) targeting two neuronal populations (indicated by either red or violet arrows). Red and violet horizontal arrows specify the target neurons of the two stimulations. Before stimulation (50 time steps), the network is in a state of synchronised self-organisation. During the stimulation, synchronised activity is present only within the two subpopulations of neurons. After stimulation has ended (500 time steps), activity remains dissociated between the two neuron ensembles, as a consequence of changes in synaptic strength. These changes are emphasised in the top right panel, showing adaptation of synaptic precision (y-axis) as each synapse (x-axis) over epochs (starting from the upper left) of one typical neuron. When changes in synaptic gain are sufficient, the synaptic structure is modified. Synaptic pruning is evident in the bottom left panel, showing the connectivity (i.e., the adjacency matrix) among all neurons, for each epoch, starting from the upper left. Stimulation of the network starts from the third epoch and lasts until the twentieth. The bottom right panel shows the free energy of each neuron (coloured lines) and the average free energy (bold blank line), over a sliding window of about 70 time steps. The green area indicates the relatively long time periods needed to induce short-term plasticity to bring about sufficient decreases in synaptic efficacy to cause pruning. The blue area highlights the free energy decrease due to the structure learning and synaptic regression.

Finally, we administered two asynchronous stimulations, targeting different pools of neurons over a prolonged period of time (approximately from 100 to 1000 ms.) – to test the capacity of the network to assimilate structured information into its synaptic connectivity and dynamics. This process is similar to unsupervised learning (e.g., blind source separation) observed both in experimental and theoretical treatments of *in vitro* neuronal cultures^60^. Typical results of this simulation are shown in Fig. 6. The key parameter here is the threshold for the Bayes factor (set at 2.5), which specifies whether a synapse is removed (i.e., pruned). This factor corresponds to confidence of approximately 90% that the log evidence (i.e., free energy) would improve in the absence of a potentially redundant synapse. The persistent stimulation causes two neuronal populations to fire in synchrony, with different phase. As a result, neurons slowly adapt the gain of their connections to the new dynamics (an example from one neuron is shown in the upper right panel in Fig. 6), until the very structure of the synaptic arborisation is modified, to simulate – from the point of view of each neuron – a change in the statistical structure of its external milieu.

In summary, these simulations offer a proof of concept that canonical aspects of neuronal dynamics and plasticity are emergent properties under variational free energy minimisation. This emergence is quite sensible to initial conditions such as number of neurons and other model parameters. This has to be expected, given the simplicity of the neuronal model adopted here, which allows us to address the fundamental link between free energy minimisation and emergent properties in neural networks, under minimal assumptions. Crucially, this minimisation is with respect to fast dynamics, fluctuations in synaptic efficacy and the very structure of network architectures; leading to generalised synchronisation, desynchronisation following stimulation and the emergence of functional specialisation.

## Discussion

We simulated neurons as systems that engage in active inference, cast in terms of variational free energy minimisation. Here, neurons are not seen as systems merely reacting to external inputs, but as active agents who influence their surroundings, based on prior beliefs about the evolution of the latter. This affords a new interpretation of neuronal dynamics. For instance, the computational implications of the neuronal response modulation caused by the history of recent activity – due, for example, to slow current dynamics^40^ or threshold adaptation^61^ – can now be read in terms of how prior beliefs dictate the effect of past observations (via recurrent connections implicit in the transition matrices). An interesting implication is that differences in self-modulation, observed along cortical layers^62^, might be understood in terms of differences in the prior beliefs of each neuron or lamina specific population.

Similarly, short-term plasticity can be interpreted, in a computational sense, as an internally generated, fast deployment of attentional resources; whereby attentional allocation depends on the extent to which EPSPs carry precise information about the network state. In the same way, these expectations influence how the model’s complexity reduction (i.e., synaptic pruning) operates. These conclusions are consistent with preceding treatments that have addressed similar issues, but at the whole-brain level. For example, the way in which short-term plasticity is conceived here instantiates an attentional mechanism analogous to precision weighting (attentional gain) at a psychological level^63^. Similarly, synaptic pruning on single neurons follows the same principles that guide complexity reduction and structural learning during sleep^64^ and online data assimilation^37^. Lastly, it is interesting to note that, in the same way the brain infers how to act upon the world^34,65^, a neuron exerts an influence on the network that leads to the realisation of its expectation. The resulting scale-free nature of neuronal dynamics stems from the fact that everything can be formulated as an aspect of variational free energy minimisation.

Casting neurons as free energy minimising systems moves the focus from externally to internally generated neuronal dynamics (i.e., intracellular belief updating). On this view, a central role is played by prior beliefs that neurons hold about their environment. As neurons actively gather evidences for their generative model, a neuronal network inevitably evolves in a way that reflects the interplay between different prior expectations; namely, that to be a neuron means to participate in a network that has an attracting set or orbit. Additional emergent properties will clearly depend on the nature of the generative models that are coupled. This speaks to the notion of investigating neuronal dynamics under different or more complex internal models. For example, neurons could have expectations about the average spiking activity (c.f., rate coding), instead of expectations about timing and history (c.f., time coding)^66,67^. Another interesting extension of the present work could be to link structure learning and synaptic regression to neurotrophic factors^68^, which constrain and shape neuronal communication in the real brain. Here we have assumed that a neuron treats inputs as serially uncorrelated and independent. Although this is sufficient to illustrate how synchronisation emerges from ensemble inference, a neurobiologically more interesting extension (noted by one of our reviewers) would be to include interactions and temporal dependencies among presynaptic inputs in the generative model: for example, by extending the simulation to multi-compartmental models, whereby dendritic components would expect to see particular input sequences, generating nonlinear, slow depolarising [Ca^2+^] dynamics; as in^31^.

Finally, the hierarchical organisation of the brain speaks to a more delicate connectivity structure than simulated above^23,57^. However, it is interesting to note that the reorganisation of the connectivity following Bayesian model reduction or pruning already entails the emergence of a primitive form of topology, which ultimately reflects the causal structure of the world (in this case the two sources of input)^60^. In neurobiology, this sort of segregation provides large-scale organising principles for the brain. For example, the segregation of ‘what’ and ‘where’ pathways in visual hierarchies^25,69^.

In summary, the free energy principle implies a coupling between the internal and external states of a system that is symmetric: a system will adapt to its environment, while changing it through action. It follows that individual priors collectively shape macroscopic dynamics, reflecting a state of synchronisation among neurons. This synchronisation is therefore a hallmark of self-organising systems, where the state of each constituent depends, perhaps indirectly, upon every other component. Despite the oversimplifications in our simulations, our *in silico* neuronal ensemble displays fundamental behaviours seen in real neuronal networks, which can be summarised as the ability to maintain a state of self-organised synchronisation in face of external perturbation.

## Methods

### Variational message passing

Variational free energy, Eq. (1), is the difference between an energy; namely, the surprise about hidden states and parameters under a posterior belief and the entropy of these beliefs. This free energy functional constitutes an upper bound on surprise (or negative log model evidence) by Jensen’s inequality:

3 $$F=-\,{{\rm{E}}}_{Q}[\mathrm{ln}\,P(\tilde{o},\tilde{s},\zeta |m)]+{{\rm{E}}}_{Q}[\mathrm{ln}\,Q(\tilde{s},\zeta )]\ge -\,\mathrm{ln}\,P(\tilde{o}|m).$$

Here $\tilde{s}=({s}_{1},{s}_{2},\ldots ,{s}_{t})$ and $\tilde{o}=({o}_{1},{o}_{2},\ldots ,{o}_{t})$ denote sequences of hidden states and observations respectively; and *ζ* is a precision or inverse temperature parameter associated with a likelihood or *A* matrix, such that

4 $$\begin{array}{c}P({o}_{\tau }^{i}|{s}_{\tau }=h,{\zeta }^{i})=\frac{{({A}^{i})}^{{\zeta }^{i}}}{{\sum }_{j}\,{({A}_{j,h}^{i})}^{{\zeta }^{i}}}\cdot \end{array}$$

This likelihood constitutes, with prior beliefs, $P(\tilde{s},\zeta |m)$ the generative model: $P(\tilde{o},\tilde{s},\zeta |m)=P(\tilde{o}|\tilde{s},\zeta )P(\tilde{s},\zeta |m)$.

Using a mean-field approximation, it is possible to factorise posterior beliefs $$Q\equiv Q(\tilde{s})Q(\zeta )={\prod }_{\tau =1}^{t}$$$$Q({s}_{\tau })\,{\prod }_{i=1}^{k}Q({\zeta }^{i})$$, by leveraging the approximate Markovian property of the state transitions $$P({s}_{\tau }|{s}_{\tau -1},\ldots ,{s}_{1},{B}_{\tau -1})\approx P({s}_{\tau }|{s}_{\tau -1},{B}_{\tau -1}({\mathscr{T}}(\tau )))$$, for $${\mathscr{T}}(\tau )=\{\tau -{t}_{{\rm{\max }}},\tau -{t}_{{\rm{\max }}}+1,\ldots ,\tau -{t}_{{\rm{\min }}}\}$$, and the likelihood mapping $$P({o}_{\tau }|{s}_{\tau },\zeta )={\prod }_{i=1}^{k}P({o}_{\tau }^{i}|{s}_{\tau },{\zeta }^{i})$$, where *k* is the number of synapses. The variational free energy associated with beliefs about states at time *τ* can then be expressed as:

5 $$\begin{array}{rcl}F(1) & = & -{{\rm{E}}}_{Q}[{\sum }_{i=1}^{k}\mathrm{ln}\,P({o}_{1}^{i}|{s}_{1},{\zeta }^{i})+\,\mathrm{ln}\,P({s}_{1}|D)-\,\mathrm{ln}\,Q({s}_{1})]\\ F(\tau ) & = & -{{\rm{E}}}_{Q}[{\sum }_{i=1}^{k}\mathrm{ln}\,P({o}_{\tau }^{i}|{s}_{\tau },{\zeta }^{i})+\,\mathrm{ln}\,P({s}_{\tau }|{s}_{\tau -1},{B}_{\tau -1}({\mathscr{T}}(\tau )))-\,\mathrm{ln}\,Q({s}_{\tau })]\,{\rm{for}}\,2\le \tau \le t\end{array}$$

The total variational free energy is given by the sum of the (time-dependent) free energy over $\tau =1,\ldots ,t$ plus a complexity regarding posterior beliefs about (time-invariant) parameters:

6 $$\begin{array}{rcl}F & = & {\sum }_{\tau =1}^{t}{{\rm{E}}}_{Q(\zeta )}[F(\tau )]+{\sum }_{i=1}^{k}{{\mathscr{D}}}_{{\rm{KL}}}[Q({\zeta }^{i})||P({\zeta }^{i})]\\  & = & -{{\rm{E}}}_{Q}[\mathrm{ln}\,P({s}_{1}|D)+{\sum }_{\tau =2}^{t}\mathrm{ln}\,P({s}_{\tau }|{s}_{\tau -1},{B}_{\tau -1}({\mathscr{T}}(\tau )))\\  &  & +\,{\sum }_{\tau =1}^{t}\{{\sum }_{i=1}^{k}\mathrm{ln}\,P({o}_{\tau }^{i}|{s}_{\tau },{\zeta }^{i})-\,\mathrm{ln}\,Q({s}_{\tau })\}+{\sum }_{i=1}^{k}\{\,\mathrm{ln}\,P({\zeta }^{i})-\,\mathrm{ln}\,Q({\zeta }^{i})\}].\,\end{array}$$

The derivative (or the first variation) of free energy with respect to beliefs about hidden states at a particular time is:

7 $$\begin{array}{rcl}\frac{\delta F}{\delta Q({s}_{\tau })} & = & -{{\rm{E}}}_{Q({s}_{\tau -1})Q({s}_{\tau +1}){\prod }_{i=1}^{k}Q({\zeta }^{i})Q({B}_{\tau })Q({B}_{\tau -1})}[\sum _{i=1}^{k}\,\mathrm{ln}\,P({o}_{\tau }^{i}|{s}_{\tau },{\zeta }^{i})+\,\mathrm{ln}\,P({s}_{\tau +1}|{s}_{\tau },{B}_{\tau })\\  &  & +\,\mathrm{ln}\,P({s}_{\tau }|{s}_{\tau -1},{B}_{\tau -1})-\,\mathrm{ln}\,Q({s}_{\tau })]\end{array}$$

Solving for zero gives the optimal posterior belief. Using model parameters and expectations, and omitting constants, this becomes:

8 $$\mathrm{ln}\,{{\bf{s}}}_{\tau }={{\boldsymbol{\zeta }}}^{1}\,\mathrm{ln}\,{{\bf{A}}}^{1}\cdot {o}_{\tau }^{1}+\ldots +{{\boldsymbol{\zeta }}}^{k}\,\mathrm{ln}\,{{\bf{A}}}^{k}\cdot {o}_{\tau }^{k}+\,\mathrm{ln}\,{{\bf{B}}}_{\tau }\cdot {{\bf{s}}}_{\tau +1}+\,\mathrm{ln}\,{{\bf{B}}}_{\tau -1}{{\bf{s}}}_{\tau -1}.$$

Here the posterior beliefs are denoted by the expectation, $Q({s}_{\tau })\equiv {{\bf{s}}}_{\tau }\in [0,1]$ and $Q({\zeta }^{i})\equiv {{\boldsymbol{\zeta }}}^{i}\in [0,1]$. Finally, we express the variational solution in terms of a gradient descent, where the free energy gradient is equal to an error term, which is the difference between the current belief and the solution given the Markov blanket:

9 $$\begin{array}{c}{\dot{{\boldsymbol{\upsilon }}}}_{\tau }=-\,{\varepsilon }_{\tau }=-\,{\nabla }_{{{\bf{s}}}_{\tau }}F\\ {{\bf{s}}}_{\tau }=\sigma ({\boldsymbol{\upsilon }})\\ {\varepsilon }_{{\boldsymbol{\tau }}}=\,\mathrm{ln}\,{{\bf{s}}}_{\tau }-({{\boldsymbol{\zeta }}}^{1}\,\mathrm{ln}\,{{\bf{A}}}^{1}\cdot {o}_{\tau }^{1}+\ldots +{{\boldsymbol{\zeta }}}^{k}\,\mathrm{ln}\,{{\bf{A}}}^{k}\cdot {o}_{\tau }^{k}+\,\mathrm{ln}\,{{\bf{B}}}_{\tau }\cdot {{\bf{s}}}_{\tau +1}+\,\mathrm{ln}\,{{\bf{B}}}_{\tau -1}{{\bf{s}}}_{\tau -1}).\end{array}$$

### Precision update (short-term synaptic plasticity)

Assuming that the priors and posteriors over precision parameters are gamma distributions^63^:

10 $$\begin{array}{lll}P({\zeta }^{i}) & \propto  & {\beta }_{z}^{i}{e}^{-{\beta }_{z}^{i}{\zeta }^{i}}\\ Q({\zeta }^{i}) & \propto  & {{\boldsymbol{\beta }}}_{z}^{i}{e}^{-{{\boldsymbol{\beta }}}_{z}^{i}{\zeta }^{i}}.\end{array}$$

Note that the posterior expectation is given by ${{\rm{E}}}_{Q({\zeta }^{i})}[{\zeta }^{i}]={{\boldsymbol{\zeta }}}^{i}={({{\boldsymbol{\beta }}}_{z}^{i})}^{-1}$. It is therefore possible to express the free energy in terms of parameters of the model (omitting constants with respect to *ζ*^*i*^),

11 $$\begin{array}{rcl}F & = & \sum _{i=1}^{k}\,{{\rm{E}}}_{Q({\zeta }^{i})}[-\sum _{\tau =1}^{t}\,{{\rm{E}}}_{Q({s}_{\tau })}[\mathrm{ln}\,P({o}_{\tau }^{i}|{s}_{\tau },{\zeta }^{i})]-\,\mathrm{ln}\,P({\zeta }^{i})+\,\mathrm{ln}\,Q({\zeta }^{i})]\\  & = & \sum _{i=1}^{k}\,{{\boldsymbol{\zeta }}}^{i}\cdot \{-\sum _{\tau =1}^{t}\,{{\bf{s}}}_{\tau }\cdot \,\mathrm{ln}\,{\bar{{\bf{A}}}}^{i}\cdot {o}_{\tau }^{i}+{\beta }_{z}^{i}{{\boldsymbol{\zeta }}}^{i}-\,\mathrm{ln}\,{\beta }_{z}^{i}+\,\mathrm{ln}\,{{\boldsymbol{\beta }}}_{z}^{i}\}\end{array}$$

Here ${\bar{{\bf{A}}}}_{hj}=\frac{{{\bf{A}}}_{hj}^{{\boldsymbol{\zeta }}}}{{\sum }_{l}\,{{\bf{A}}}_{lj}^{{\boldsymbol{\zeta }}}}$ denotes a normalised (i.e., probability) matrix. Here, we are interested in the precision of the likelihood matrix, but the update equations for the precision of the transition matrix are the same. Taking the derivative of free energy with respect to the expected precision gives:

12 $$\begin{array}{c}\frac{\partial F}{\partial {{\boldsymbol{\zeta }}}^{i}}=-\,\sum _{\tau =1}^{t}\,{{\bf{s}}}_{\tau }\cdot {\partial }_{{{\boldsymbol{\zeta }}}^{i}}\,\mathrm{ln}\,{\bar{{\bf{A}}}}^{i}\cdot {o}_{\tau }^{i}+{\beta }_{z}^{i}-{{\boldsymbol{\beta }}}_{z}^{i}.\end{array}$$

The update equation for sensory precision is then:

13 $$\begin{array}{c}{{\boldsymbol{\beta }}}_{z}^{i}={\beta }_{z}^{i}-\sum _{\tau =1}^{t}\,{{\bf{s}}}_{\tau }\cdot {\partial }_{{{\boldsymbol{\zeta }}}^{i}}\,\mathrm{ln}\,{\bar{{\bf{A}}}}^{i}\cdot {o}_{\tau }^{i}.\end{array}$$

We express the variational solution in terms of gradient descent, whereby

14 $$\begin{array}{c}{\dot{{\boldsymbol{\beta }}}}_{z}^{i}={\varepsilon }_{z}^{i}\,;\,{{\boldsymbol{\zeta }}}^{i}={({{\boldsymbol{\beta }}}_{z}^{i})}^{-1}.\end{array}$$

Here, the error term corresponds to:

15 $$\begin{array}{c}{\varepsilon }_{z}^{i}={\beta }_{z}^{i}-\sum _{\tau =1}^{t}\,{{\bf{s}}}_{\tau }\cdot {\partial }_{{{\boldsymbol{\zeta }}}^{i}}\,\mathrm{ln}\,{\bar{{\bf{A}}}}^{i}\cdot {o}_{\tau }^{i}-{{\boldsymbol{\beta }}}_{z}^{i},\end{array}$$

where the partial derivative can be written as:

16 $$\begin{array}{rcl}{{\bf{s}}}_{\tau }\cdot {\partial }_{{{\boldsymbol{\zeta }}}^{i}}\,\mathrm{ln}\,{\bar{{\bf{A}}}}^{i}\cdot {o}_{\tau }^{i} & = & \sum _{j=0,1}\,\mathrm{ln}\,{{\bf{A}}}_{\cdot ,j}^{i}\cdot {\varepsilon }_{o,\tau }^{i,j}\\ {\varepsilon }_{o,\tau }^{i,j} & = & ({o}_{\tau }^{i}-{{\bar{{\bf{A}}}}^{i}}_{\cdot ,j}){{\bf{s}}}_{\tau ,j}.\end{array}$$

These are the equations used in Fig. 2 to illustrate the synaptic gain update equations.

### Bayesian Model Reduction (synaptic pruning)

We compare the approximate log evidence or free energy of a reduced model $\tilde{P}(o)$ and the full model P(*o*), where the two are identical except for the fact that the former is equipped with an infinitely precise prior centred on zero for a subset of parameters. Here, *θ* corresponds to a set of precisions $\theta =\zeta =({\zeta }^{1},\ldots ,{\zeta }^{k})$. The calculation of the Bayes factor or difference in free energy is as follows:

17 $$\begin{array}{ccc}\mathop{P}\limits^{ \sim }(o)\approx P(o)\int Q(\theta )\frac{\mathop{P}\limits^{ \sim }(\theta )}{P(\theta )}d\theta  & \Rightarrow  & {\rm{l}}{\rm{n}}\,\mathop{P}\limits^{ \sim }(o)\approx \,{\rm{l}}{\rm{n}}\,P(o)+\,{\rm{l}}{\rm{n}}\,Z\\ Z & = & {{\rm{E}}}_{Q}[\frac{\mathop{P}\limits^{ \sim }(\theta )}{P(\theta )}]\\ {\rm{\Delta }}F & = & {\rm{l}}{\rm{n}}\,Z\end{array}$$

We assume priors over parameters describing a gamma distribution^63^, with *α* and *β* being the shape and rate parameter respectively. If we set *α* = 1 for both reduced and full models, then $E[\zeta ]=\frac{1}{\beta }$ is the expected value of the synaptic precision parameter. The derivation of the Bayes factor is then:

18 $$\begin{array}{c}P(\theta )={\rm{\Gamma }}(1,\beta )\\ \tilde{P}(\theta )={\rm{\Gamma }}(1,\tilde{\beta })\\ Q(\theta )={\rm{\Gamma }}(1,{\boldsymbol{\beta }})\\ {\rm{\Gamma }}(1,\beta )=\frac{\beta {e}^{-\beta \theta }}{{\rm{\Gamma }}(1)}\\ \mathrm{ln}\,Z=\,\mathrm{ln}\,\tilde{\beta }-\,\mathrm{ln}\,\beta +\,\mathrm{ln}\,{{\rm{E}}}_{Q}[{e}^{(\beta -\tilde{\beta })\theta }]\\ {E}_{Q}[{e}^{(\beta -\tilde{\beta })\theta }]=\frac{{\boldsymbol{\beta }}}{{\boldsymbol{\beta }}-\beta -\tilde{\beta }}\\ \begin{array}{c}{\rm{\Delta }}F=ln\tilde{\beta }-ln\beta +ln\beta -ln({\boldsymbol{\beta }}-\beta +\tilde{\beta })\end{array}\end{array}$$

The last equality specifies the difference in variational free energy or log Bayes Factor between the reduced and full model. Neurons use this difference, calculated *post hoc* (at the end of the epoch *e*), to decide whether to prune or retain a specific parameter or synapse.

## Data Availability

All relevant data are within the paper. MATLAB source codes are appended as Supplementary Source Codes.
